# Comparative Lipidomic Profiling of *S. cerevisiae* and Four Other Hemiascomycetous Yeasts

**DOI:** 10.3390/metabo2010254

**Published:** 2012-03-02

**Authors:** Eva-Maria Hein, Heiko Hayen

**Affiliations:** Leibniz-Institut für Analytische Wissenschaften-ISAS-e.V., Otto-Hahn-Str. 6b, D-44227 Dortmund, Germany

**Keywords:** yeast strains, *Saccharomyces cerevisiae*, *Saccharomyces bayanus*, *Kluyveromyces thermotolerans*, *Pichia angusta*, *Yarrowia lipolytica*, glycerophospholipid profiling, LC-MS, lipidomics

## Abstract

Glycerophospholipids (GP) are the building blocks of cellular membranes and play essential roles in cell compartmentation, membrane fluidity or apoptosis. In addition, GPs are sources for multifunctional second messengers. Whereas the genome and proteome of the most intensively studied eukaryotic model organism, the baker’s yeast (*Saccharomyces cerevisiae*), are well characterized, the analysis of its lipid composition is still at the beginning. Moreover, different yeast species can be distinguished on the DNA, RNA and protein level, but it is currently unknown if they can also be differentiated by determination of their GP pattern. Therefore, the GP compositions of five different yeast strains, grown under identical environmental conditions, were elucidated using high performance liquid chromatography coupled to negative electrospray ionization-hybrid linear ion trap-Fourier transform ion cyclotron resonance mass spectrometry in single and multistage mode. Using this approach, relative quantification of more than 100 molecular species belonging to nine GP classes was achieved. The comparative lipidomic profiling of *Saccharomyces cerevisiae*, *Saccharomyces bayanus*, *Kluyveromyces thermotolerans*, *Pichia angusta*, and *Yarrowia lipolytica* revealed characteristic GP profiles for each strain. However, genetically related yeast strains show similarities in their GP compositions, e.g., *Saccharomyces cerevisiae* and *Saccharomyces bayanus.*

## 1. Introduction

Lipidomic profiling methods reflect the lipid status of a phenotype at a particular time point [1,2,3] and are therefore valuable tools to improve the understanding the biological roles of lipids. Unlike genes or proteins, the lipid composition can rapidly be influenced by external factors like nutrition or environmental conditions and is alterable even within seconds [2,3,4,5]. It is not sufficient enough to consider these topics solely on the transcriptional level, because many genes involved in lipid biosynthesis or transport exist in multiple copies, which can compensate a dysfunction or lack in one gene and thus, no effects on the lipid profile can be observed. Moreover, several studies have shown that organisms like the yeast *S. cerevisiae* can tolerate great changes in their lipid composition, compensating for example for the absence of one lipid by overproduction of another, without notable effects on their viability [5,6].

Despite many mass spectrometry based lipidomics methods developed today [7], the current knowledge of the lipidome of eukaryotic organisms is still limited. As the lipidome of higher eukaryotic organisms consists of hundreds to thousands of individual molecular species, a model organism is needed, which possesses a relatively simple lipidome, but still reflects the main biosynthetic and metabolic pathways of higher eukaryotic organisms. It should be easy to handle and also, if necessary, easy to manipulate. Another important criterion is a detailed knowledge on gene, protein and also lipid biosynthesis, which enables to fill gaps in the understanding of a complex biological network. Such a suitable eukaryotic organism is yeast, as it fulfills all the requirements listed above [8,9].

One of the yeasts investigated best is the common bakers’ yeast, *S. cerevisiae*, for which complete genome, as well as detailed protein data, are available. Therefore, many studies have used this model organism for lipidomics studies. One of the major lipid categories of eukaryotic organisms are glycerophospholipids (GPs), which cover diverse biological roles like cell compartmentalization, energy storage and multiple signaling functions. Consequently, they are the subject of many studies, because their biosynthesis and metabolism is very similar to those of higher eukaryotes, with three main exceptions. Firstly, yeast phosphatidylserine (PS) is mainly synthesized by the CDP-DAG pathway and not by PS synthase from phosphatidylethanolamine (PE). Secondly, for phosphatidylcholine (PC) synthesis, an alternative route exists besides the Kennedy-Pathway (CDP-choline), which is the exclusive pathway in mammals. In yeast, the successive methylation of PE to *mono*-methyl-phosphatidylethanolamine (MMPE), *di*-methyl-phosphatidylethanolamine (DMPE) and finally PC occurs, catalyzed by *N*-methyl-transferases [2,9]. Thirdly, the difference to mammals is the relatively low abundance of polyunsaturated fatty acids (PUFAs), or rather the complete absence of PUFAs like in *S. cerevisiae*.

Numerous studies have been dedicated to understand the role of GPs in *S. cerevisiae*. It has been shown that the faultless biosynthesis and metabolism of particular GPs appear to be essential for cell vitality. For instance, mutations in the gene encoding the phosphatidylinositol (PI) synthase are lethal for the organism [6,9]. On the other hand, not all GP classes seem to be essential for the survivability of the organism, as defects in genes encoding enzymes involved in PC, PE or PS biosynthesis result in phenotypic changes but are not lethal under laboratory conditions. Adaptive changes of GP composition after applying varying environmental conditions (e.g., elevated growth temperatures) or by supplementation of cell culture media (e.g., with fatty acids) are also investigated in several studies along with changes in lipid profile when comparing mutants with defects in lipid biosynthesis [10,11,12].

The question addressed in this paper is if phylogenetically different yeast strains possess characteristic GP profiles, and if genetically closely related strains show analogies in their GP composition. The profiling was carried out by the previously described HPLC/LIT-FTICRMS-method [13] including automated data processing by the Profiler-Merger-Viewer software [14], of which the applicability for different complex GP samples was demonstrated recently [15,16].

## 2. Results and Discussion

**Figure 1:**
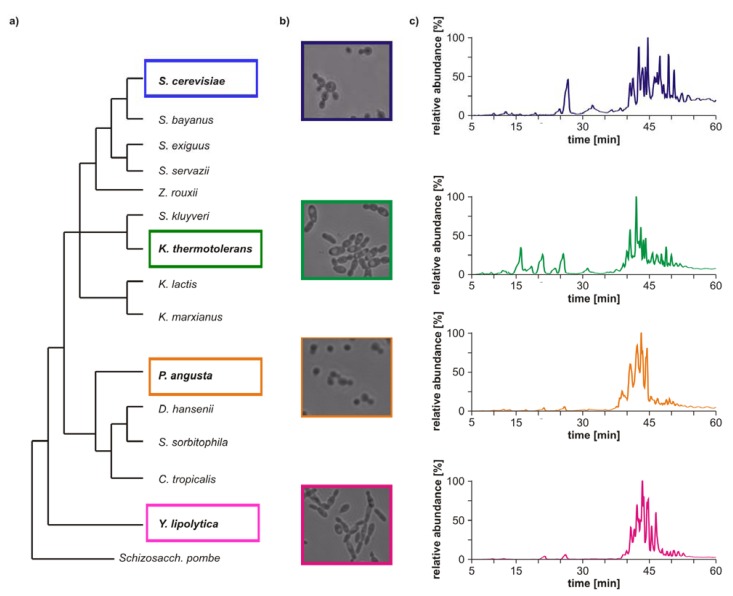
(**a**) Phylogeny of hemiascomycetous yeasts based on 15S-rRNA data (adapted from [17]); (**b**) light microscopic pictures of the chosen yeast strains and (**c**) sections of the total ion currents (TICs) of the HPLC/ESI-LIT-FTICRMS-measurements of four yeast strains.

Four yeast strains with few genetic analogies were selected to investigate possible characteristic GP profiles based on genetic differences. *Saccharomyces cerevisiae* (common baker’s yeast) was selected as the first candidate, because this organism has been investigated in detail on protein, gene as well as on lipid levels. *Kluyveromyces thermotolerans*, *Pichia angusta* and *Yarrowia lipolytica* were chosen for the comparative study, as they are not closely related to each other (see Figure 1a). A further aim of the study was to reveal analogies in the lipid profile of closely related yeast strains. Accordingly, *Saccharomyces bayanus* was chosen as a close relative of *Saccharomyces cerevisiae*. To avoid variations in the GP profiles due to changing external conditions, all strains were grown and extracted in parallel, using the same medium batch. Hence, observed characteristics are likely based on genetic differences.

Morphological differences between the four yeast strains can already be observed on a macroscopic level (Figure 1b) and even without detailed lipid profiling the total ion currents (TICs) of the HPLC/ESI-LIT-FTICRMS-measurements showed different patterns (Figure 1c). Within a yeast strain, the profile was reproducible in all biological replicates (n = 3; data not shown).

**Figure 2:**
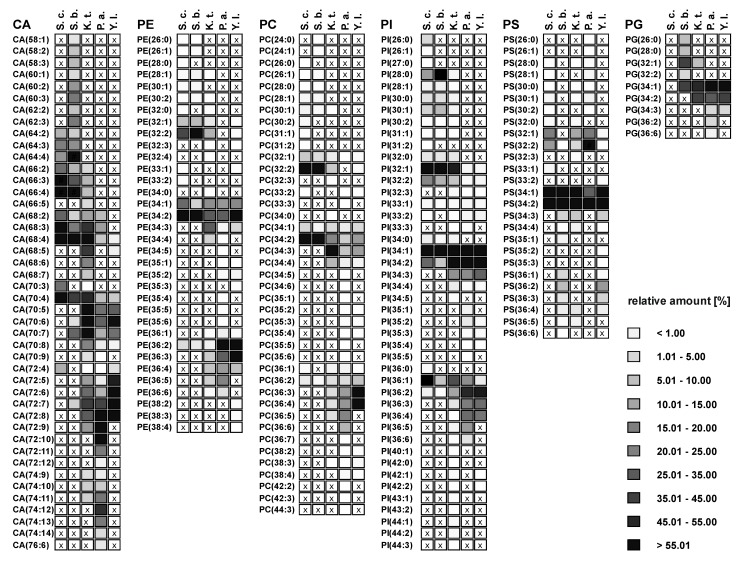
Overview of species distribution in the six most common GP classes: cardiolipins (CA), phophatidylethanolamines (PE), phosphatidylcholines (PC), phosphatidylinositoles (PI), phosphatidylserines (PS), and phosphatidylglycerols (PG) for *S. cerevisiae (S.c.)*, *S. bayanus (S.b.)*, *K. thermotolerans (K.t.)*, *P. angusta (P.a.)* and *Y. lipolytica (Y.l.)*. (x indicate not detected GP species).

A detailed insight into the complexity of the GP profiles was realized by HPLC/MS(/MS) investigation of the lipid extracts. Using the recently developed aforementioned HPLC/ESI-LIT-FTICRMS method, 106–120 molecular GP species from nine GP classes (cardiolipins (CA), phosphatidic acids (PA), phophatidylethanolamines (PE), *mono*-methyl-phosphatidylethanolamines (MMPE), *di*-methyl-phosphatidylethanolamines (DMPE), phosphatidylcholines (PC), phosphatidylinositoles (PI), phosphatidylserines (PS), and phosphatidylglycerols (PG)) were relatively quantified in *S. cerevisiae*, *S. bayanus*, *K. thermotolerans*, *P. angusta*, and *Y. lipolytica*. A comparison of the GP species belonging to the six most abundant GP classes of the five yeast strains is given in Figure 2. Please note that fatty acid chains are abbreviated (xx:y), with xx the total number of carbon atoms and y the sum of double bonds in the fatty acid chains. The relative amount of one species is calculated in relation to the sum of all species contributing to the same GP class.

These comparative GP profiles show that significant differences in number, distribution and relative amount of the identified GP species exist among the phylogenetically different yeast strains. In general, the number of identified species is less in *S. cerevisiae* and *S. bayanus*, whereas *K. thermotolerans*, *P. angusta* and *Y. lipolytica* possess a larger variety of GP species. In addition, the number, as well as the distribution, of major GP species is significantly different in the genetically diverse yeasts, whereas the patterns of the related yeasts strains show analogies. In particular, *S. cerevisiae* and *S. bayanus* possess in general four major species, with rather short acyl chains and a lower number of double bonds. The latter are PE(32:2), PE(34:2), PC(32:2) and PC(34:2), respectively. The yeast *Y. lipolytica* possesses also only a few major species in each GP class, but unlike the *Saccharomyces* strains, the chain length and degree of unsaturation is considerably higher. In contrast, the lipid profiles of *K. thermotolerans* and *P. angusta* show a larger variety of GP species in each class. Compared to the three previous yeast strains, the fatty acid chains are longer and have an increased number of double bonds. Compared to each other, this trend is stronger in *P. angusta*. All these differences are especially pronounced in the class of CAs (Figure 2). An exception from this divergence seems to be in the GP classes PS, PI and PG. The major species identified in these classes are very similar for all investigated yeast strains (Figure 2).

**Figure 3:**
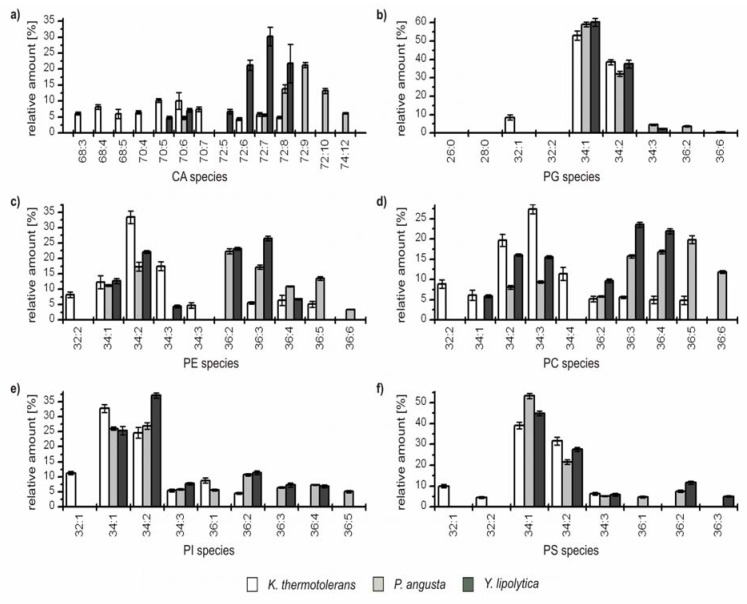
Relative amounts of the GP species within the six major GP classes (**a**) cardiolipins (CA); (**b**) phosphatidylglcerols (PG); (**c**) phophatidylethanolamines (PE); (**d**) phosphatidylcholines (PC); (**e**) phosphatidylinositoles (PI); and (**f**) phosphatidylserines (PS) for *K. thermotolerans* (□), *P. angusta* (■) and *Y. lipolytica* (■). The relative amount of respective species is referred to 100% peak area of all identified GPs within one class and yeast strain. Only species which contributed to more than 5% of the respective GP class are displayed.

For a more detailed interpretation, the yeast strains were divided into two groups based on the overall GP pattern. The first group comprises *K. thermotolerans*, *P. angusta* and *Y. lipolytica*, the second group contains *S. cerevisiae* and *S. bayanus*. The relative amounts of the identified species from the first group are depicted in Figure 3. For better representation, only species which contributed at least to 5% to the GP profile of a single class are represented (an overview of all identified GPs and their relative amounts is given in Table S1 of the Supporting Information).

From this perspective, characteristic differences within the group can be recognized. The major GP species of *K. thermotolerans* have 34 carbon atoms in their fatty acid residues and one to three double bonds. Some less abundant species containing 32 carbon atoms were identified in all GP classes. In addition, species with 36 carbon atoms and up to five double bonds could be identified in the classes of PE, PC and PI. In contrast, *P. angusta* shows a narrower distribution, exhibiting major species with 34 and 36 carbon atoms, and is thus shifted by one or two C_2_-units compared to *K. thermotolerans*. The number of double bonds varies from one to six. This distribution is also observed within the PEs. A slightly asymmetric shift to species with 72 carbon atoms for CA and 36 carbon atoms is observed for PC (Figure 3a,d), whereas the classes of PG, PI and PS show a shift to species with 34 carbon atoms (Figure 3b, e and f). The species distribution of *Y. lipolytica* is similar to that of *P. angusta* concerning the number and shift of carbon atoms, but in contrast, the maximum number of double bonds observed was four. In all three yeast strains, minor amounts of odd numbered GP species were identified, consisting mainly of 33 and 35 carbon atoms and more seldom of 31 carbon atoms, with a maximum relative amount of 1%. The impressions of Figure 2 are confirmed, as the species distribution of CA, PE and PC is particularly characteristic for each of the three yeast strains, whereas the profiles of PG, PI and PS possess identical major species and just small differences in the less abundant species are observed.

Analysis of the compositions of GPs based on HPLC/ESI-LIT-FTICRMS in multistage mode showed that palmitic acid (16:0) and stearic acid (18:0) were the most prominent fatty acid residues, whereas palmitoleic acid (16:1) and oleic acid (18:1) were the major unsaturated species. Minor species comprised FAs 14:0, 14:1, 12:0 and 12:1 as well as the odd numbered FAs 15:0, 15:1, 17:0 and 17:1. These results are in good agreement with additional GC/MS measurements after hydrolysis and derivatization of the lipid extracts (data not shown).

**Figure 4:**
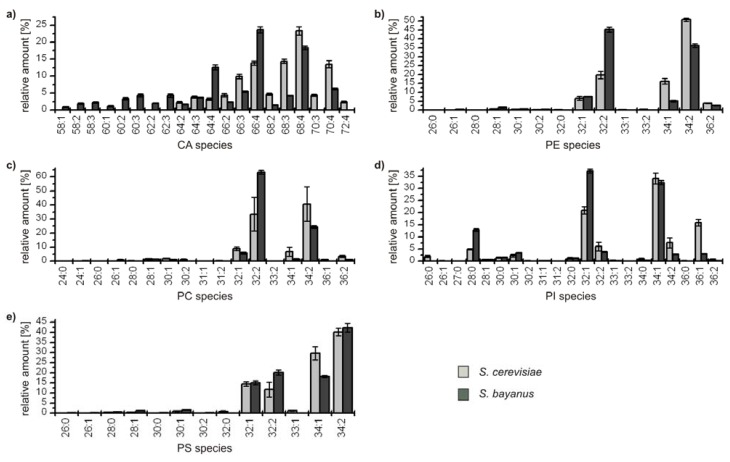
Relative amounts of the GP species within the classes (**a**) cardiolipins (CA); (**b**) phophatidylethanolamines (PE); (**c**) phosphatidylcholines (PC); (**d**) phosphatidyl­inositoles (PI); and (**e**) phosphatidylserines (PS) for *S. cerevisiae* (■) and *S. bayanus* (■). The relative amount of respective species is referred to 100% peak area of all identified GPs within one class and yeast strain.

The detailed comparative profiling of the second group reveals the similarity of the genetically related yeasts *S. cerevisiae* and *S. bayanus*, respectively. All identified species are considered due to the lower complexity of the profiles, and the results are depicted in Figure 4.

In all classes the distribution of the major compounds of *S. cerevisiae* and *S. bayanus* is similar, showing always four major GP species with 32 and 34 carbon atoms and one to two double bonds, except for the class of CA, which possesses a broader distribution due to its variation possibilities based on four bound fatty acids. It has to be noted that in the classes of PE and PC the most abundant species GP(34:2) of *S. cerevisiae* is the second most abundant species of *S. bayanus*, and vice versa for the species GP(32:2). Concerning the minor components, the lipid profiles of both yeast strains also show remarkable analogies. Only few species with 36 carbon atoms in the acyl chains could be identified and also some odd numbered species, bearing 31 and 33 carbon atoms. The maximum number of double bonds for a GP-species was two, except for CAs, where minor species with up to seven double bonds were identified. As PGs only were identified for *S. bayanus*, the diagram is not shown. The main species identified were PG(32:1), 40.8% ± 1.6% and PG(34:1), 43.1% ± 2.2%. Furthermore, PG(28:0), PG(26:0) and PG(32:2), contributing 7.7% ± 0.9%, 5.6% ± 0.8% and 2.8% ± 0.9% were identified.

The GP profiling data obtained for *S. cerevisiae* within this work are in large parts in good agreement with previously published data by Ejsing *et al.* [11], who used a quantitative shotgun mass spectrometric approach. In both studies the same major GP species were identified, in particular PE(32:1), PE(32:2), PE(34:1), PE(34:2), PC(32:1), PC(32:2), PC(34:1), PC(34:2), PI(28:0), PI(32:1), PI(32:2), PI(34:1), PI(34:2), PS(32:1), PS(32:2), PS(34:1), and PS(34:2). Different results, referring to the study of Ejsing *et al.* were for example obtained for the class of CAs. The major species identified in this study were CA(66:3), CA(66:4), CA(68:3), CA(68:4) and CA(70:4), whereas Ejsing *et al.* found the species CA(64:4), CA(66:2) beside CA(66:4) and CA(68:4). In addition, no phosphatidic acid (PA) could be detected in *S. cerevisiae* in contrast to the findings of Ejsing *et al.*, and each positive Profiler-Merger-Viewer software hit turned out to be an in-source fragmentation artifact of the corresponding PS species. However, the fact that PAs could be unambiguously identified in the other yeast strains, demonstrates that this class can also be detected by the applied method (see Table S1 of the Supporting Information), but PAs may be below the limits of detection in the case of *S. cerevisiae*. In addition to these predominant GP classes, in both studies minor amounts of PG could be identified, as well as the *lyso*-forms of PE, PC, and PI. Moreover, the intermediates of PC biosynthesis via the PE-methylation pathway were identified, *i.e.* MMPE and DMPE. 

These results are also confirmed by other studies, where FA(16:1), FA(16:0) and FA(18:1) are described as most abundant fatty acids linked to the GPs. However, also FA(18:0) and FA(14:0) were frequently reported, which is in good accordance with the study of Ejsing *et al.* and further former studies [4,9,10,11,17,18]. The instance that exclusively saturated and mono-unsaturated fatty acids were identified in *S. cerevsisae* in the GP classes bearing two FAs is further corroborated by the genetic data of fatty acids synthesis, as *S. cerevisiae* owns only a single fatty acid desaturase, *ole1p*, which introduces a double bond in Δ9 position of FAs [4,19,20]. Therefore, the restricted capacity of introducing double bonds is reflected in the GP profile of *S. cerevisisae*.

Interestingly, *Y. lipolytica* is known to possess an additional FA desaturase, which introduces a double bond in Δ12 position [19]. This can explain the higher number of double bonds found in *Y. lipolytica’s* GPs species. Unfortunately, no genetic data are available for the remaining yeast strains. However, due to the mentioned agreement of existing genetic data with the obtained lipid profiles, some of the lacking gene data can be extrapolated from the lipid profiles. *S. bayanus* also seems to possess only the Δ9 desaturase, analogous to *S. cerevisiae*. *K. thermotolerans* and *P. angusta* should own the additional Δ12 desaturase, like *Y. lipolytica*. Moreover, they may contain further desaturases, introducing a third and fourth double bond in the FAs, as GPs with more than four double bonds in both fatty acid residues are identified for *K. thermotolerans* and *P. angusta*. For other fungi, the existence of those desaturases, which are likely Δ15 and Δ17 desaturases, is reported [21,22,23,24,25,26,27,28,29,30]. An external source, for example from the culture medium, can be excluded, as minimal medium was used [4].

Besides the differences in the identity of the GP species, their relative amounts and distributions, we also studied the contribution of each the major GP classes to the whole GP lipidome. The relative amount was calculated as sum of all species belonging to a class in relation to the sum of all identified GPs. An overview is given in Table 1.

**Table 1:** Distribution of GP classes in the different yeasts. Relative amount is calculated as sum of all species constituting the same GP class.

GP class	*S. cerevisiae*		*S. bayanus*		*K. thermotolerans*		*P. angusta*		*Y. lipolytica*	
	average [%]	SD [%]	average [%]	SD [%]	average [%]	SD [%]	average [%]	SD [%]	average [%]	SD [%]
CA	3.6	1.6	6.1	0.6	3.1	1.2	2.4	0.4	2.8	0.3
PG	0.0	0.0	0.4	0.0	0.4	0.1	2.5	0.2	1.6	0.1
PA	not identified		0.3	0.0	< 0.1		0.5	0.1	1.3	0.1
PE	17.4	1.1	24.7	2.2	14.9	2.3	27.8	0.8	31.9	0.4
MMPE	0.3	0.0	0.1	0.0	< 0.1		2.5	0.2	0.9	0.0
DMPE	2.6	0.5	0.3	0.0	0.9	0.2	not identified		1.8	0.1
PC	31.5	8.7	12.5	3.0	26.3	7.9	40.0	0.6	32.6	0.8
PI	39.9	8.4	45.8	4.0	49.5	9.1	17.6	0.8	19.3	0.7
PS	4.8	0.5	9.7	0.7	4.9	0.7	6.7	0.3	7.7	0.3

The main GP class of *S. cerevisiae* was determined to be PI. Equal observations are reported by Ejsing *et al* [11], but are deviating from former reports [4,9,10,17,18]. PI is also the major GP class in *S. bayanus* and *K. thermotolerans.* The GP contents of *P. angusta* and *Y. lipolytica* mainly consist of PCs. It is remarkable that the closely related yeasts *S. cerevisiae* and *S. bayanus* differ significantly from each other concerning the relative amounts of PE, DMPE, PC and PS, unlike to their strong analogies in distribution of the species within a class. However, it is still obvious that the divergence in the GP class distribution is larger with increasing genetic differences.

## 3. Experimental Section

### 3.1. Chemicals and Growth Medium

Acetonitrile (ACN), methanol (MeOH) and H_2_O were of LC/MS grade, Chloroform (CHCl_3_) and *n*-propanol were of HPLC grade. All solvents were purchased from Carl Roth GmbH & Co. KG (Karlsruhe, Germany) or Sigma-Aldrich Chemie GmbH (Steinheim, Germany). The ammonium acetate (NH_4_Ac) and acetic acid (HAc) were of analytical grade and obtained from Merck KGaA (Darmstadt, Germany). Glycerophospholipid standards used were purchased from Avanti Polar Lipids (Birminghan, AL, USA) and prepared as described by Hein *et al.* [13]. Trimethylsulfonium hydroxide (0.25 M in MeOH) for derivatiziation was obtained from Macherey-Nagel (Düren, Germany). For yeast cultivation, mineral medium [31] was used, containing (per liter) 20 g D-glucose, 5.0 g (NH_4_)_2_SO_4_, 3.0 g KH_2_PO_4_, 0.5 g MgSO_4_∙7 H_2_O, 4.5 mg ZnSO_4_∙7 H_2_O, 0.3 mg CoCl_2_∙6 H_2_O, 1.0 mg MnCl_2_∙4 H_2_O, 0.3 mg CuSO_4_∙5 H_2_O, 4.5 mg CaCl_2_∙2 H_2_O, 3.0 mg FeSO_4_∙7 H_2_O, 0.4 mg NaMoO_4_∙2 H_2_O, 1.0 mg H_3_BO_3_, 0.1 g KI, 15.0 mg EDTA, 0.05 mg biotin, 1.0 mg calcium pantothenate, 1.0 mg nicotinic acid, 25.0 mg inositol, 1.0 mg pyridoxine, 0.2 mg *p*-aminobenzoic acid and 1.0 mg thiamine. To avoid pH changes due to ammonia uptake and acetate production, the medium was supplemented with 50 mM potassium hydrogen phthalate. All chemicals for the mineral medium were purchased from Fluka Chemie AG (Buchs, Switzerland) at the highest purity available.

### 3.2. Yeast Strain, Cultivation and Lipid Extraction

The hemiascomycetous yeasts used in this study were the wild-type strains of the Génolevures project (http://cbi.labri.fr/Genolevures/index.php) [32], purchased from CLIB (Collection de levures d’intérét biotechnologique, Thiverval Grignon, France). The yeasts *S. cerevisiae* CEN.PK 113-7D, *Saccharomyces bayanus*, *Klyuveromyces thermotolerans*, *Pichia angusta* and *Yarrowia lipolytica* were grown at 30 °C in 500 mL shake-flask cultures containing 50 mL mineral medium (pH 5.0). Growth of yeasts was monitored by measurements of the optical density at a wavelength of 600 nm (OD_600_). An OD_600_ value of 1.0 correlated to a cell dry weight (CDW) of about 0.17 g_CDW_/L. Shake-flask experiments were started from overnight cultures of the respective yeasts at a cell concentration of 0.17 g_CDW_/L. Cells were harvested for lipid analysis at a biomass concentration of 1.7 g_CDW_/L (OD_600_ = 10.0). For the extraction procedure the method of Bligh and Dyer [33] was modified, omitting the use of an aqueous phase to increase the recovery of acidic GPs. Lipid extraction was carried out with 15 mg_CDW_ using the appropriate volume of culture medium. The samples were transferred to Teflon centrifuge tubes to guarantee high recovery. Cells were harvested by centrifugation (2 min, 4,000 g, 0 °C; 5702 R, Eppendorf, Hamburg, Germany). The pellet was gently washed with 5 mL of deionized H_2_O (0 °C), centrifuged again (2 min, 4,000 g, 0 °C) before resuspension in 3 mL MeOH (0 °C) to quench all metabolic processes. For the extraction of the lipids 6 mL CHCl_3_ were added. The extraction was carried out by sonication for 10 min, followed by shaking for 30 min, once more sonication for 10 min and shaking for 1 h. The cell residues were separated by centrifugation (10 min, 4,000 g, 0 °C) and the extracts were transferred to silylated 1.5 mL glass vials, dried under a nitrogen stream at 30 °C and stored at −20 °C. For analysis, the samples were reconstituted in ACN/MeOH/CHCl_3_ (49:49:2, *v/v/v*).

### 3.3. High Performance Liquid Chromatography/Mass Spectrometry

For HPLC/ESI-LIT-FTICRMS analysis buffer was added to the reconstituted sample to a final concentration of 5 mM NH_4_Ac/50 mM HAc (pH 3.75). Chromatographic separations were performed using a Surveyor MS pump and Surveyor autosampler (Thermo Fisher Scientific, San Jose, CA, USA). A 5 µL full loop injection was used. Separation was carried out on a microbore C_4_-Nucleodur Gravity column (150 mm × 1 mm i.d., particle size 5 µm) from Macherey-Nagel (Düren, Germany). The following binary gradient was used at a flow rate of 115 µL/min: 0 min 50% B, 2 min 50% B, 35 min 75% B, 45 min 100% B, 65 min 100% B, 66 min 50% B, 80 min 50% B. Mobile phase A consisted of 2.5% ACN, 2.5% MeOH and 95% H_2_O (*v/v/v*) with 5 mM NH_4_Ac/50 mM HAc (pH 3.75). Mobile phase B contained 5% H_2_O, 25% *n*-propanol, 35% ACN and 35% MeOH (*v/v/v/v*) with 5 mM NH_4_Ac/50 mM HAc (pH 3.75).

The ESI-LIT-FTICRMS experiments were carried out using a LTQ FT Fourier transform ion cyclotron resonance hybrid-mass spectrometer (Thermo Fisher Scientific, Bremen, Germany), equipped with a 7.0 T actively shielded superconducting magnet and electrospray ionization source. All analyses were carried out in negative ionization mode. The instrument was operated in the data-dependent mode. Survey centroid MS spectra in the mass range *m/z* 185–1,850 were acquired in the FTICR with a resolution R = 25,000 at *m/z* 400 (target accumulation value 5,000,000, maximal ion accumulation time 750 ms). The two most intensive ions were sequentially isolated for accurate mass measurements by a FTICR “SIM scan” in a narrow mass window (±5 Da, R = 50,000, target accumulation value 100,000, maximal ion accumulation time 750 ms) in the profile mode. Subsequent fragmentations (MS^2^, MS^3^) were carried out in the linear ion trap by collision-induced dissociation (CID; target accumulation value 10,000, maximal ion accumulation time 150 ms). Former target ions selected for MS/MS were dynamically excluded for 60 s. The total cycle time was approximately 4.6 s. The general mass spectrometric conditions were: Spray voltage: −3.5 kV, sheath gas flow: 30 arbitrary units, auxiliary gas flow: 5 arbitrary units and sweep gas flow: 2 arbitrary units. Ion transfer capillary temperature was set to 225 °C and the following parameters were used for CID MS^2^ and MS^3^ experiments: Normalized collision energy: 30%, activation: q = 0.25 and activation time: 30 ms. Ion selection thresholds were 500 counts for SIM scans, 500 counts for MS^2^ and 100 counts for MS^3^ experiments.

### 3.4. Gas Chromatography/Mass Spectrometry

An aliquot (100 µL) of the reconstituted sample was transferred to a silylated HPLC vial glass insert (200 µL) and dried under a nitrogen stream at 30 °C. 30 µL CHCl_3_ and 70 µL trimethylsulfoniumhydroxid (0.25 M in MeOH) were added, mixed thoroughly and incubated for 60 min at 60 °C. The analysis was carried out on a Focus GC coupled to a Polaris Q mass spectrometer (both Thermo Fisher Scientific, Dreieich, Germany). A HP-5 MS column (30 m; 0.25 mm i.d.; 0.25 µm film thickness; GGA, Moers, Germany) and the following temperature program were used: 150 °C (4 min), 2 °C/min, 250 °C. Sample was injected in splitless mode, injector temperature was set to 250 °C and transfer capillary temperature was 280 °C. The following mass spectrometric parameters were used: acquisition delay 3 min, ion source temperature 200 °C, full scan range *m/z* 35–500, EI = 70 eV, in positive ionization mode.

### 3.5. Data Evaluation

For conversion of raw data files into text files, the implemented file converter of Xcalibur^TM^ (Thermo Fisher Scientific) was used. These files were analyzed by the “Profiler-Merger-Viewer” software package described in detail by Hein *et al.* [14]. This application is written in Java^TM^ (Sun Microsystems) and uses Microsoft Excel^TM^ as output format.

## 4. Conclusions

In this comparative lipidomic study, the GP profiles of five phylogenetically different yeasts were investigated. The aim of the study was to answer the question as to whether these organisms possess a characteristic GP pattern and if genetic relation can also be recognized by analysis of the GP profile. Based on the HPLC/ESI-LIT-FTICRMS method and the data processing by the Profiler-Merger-Viewer software, a minimum of 106 GP species (in *S. cerevisiae*), covering nine major GP classes was relatively quantified. The study enabled a detailed insight into the species identity and distribution of relative amounts within a GP class. Moreover, the relative amounts of the GP classes in the examined yeast were also determined. The results are in good agreement with a recently published comprehensive study by Ejsing *et al.* [11]. Comparison of the five yeast strains revealed characteristic GP profiles, which were reproducible in biological replicates. Even the closely related yeast strains *S. cerevisiae* and *S. bayanus* show—notwithstanding their analogies in species identity and distribution—significant differences in the relative amount of these species. This deep insight allows the conclusion that characteristic genetic traits as well as phylogenetic relationships are reflected in the GP profile of organisms, although the lipidome describes the actual status of an organism. It has to be noted again that the basis of these results are comparable environmental conditions as well as equal nutrients. The obtained results are in accordance with existing genomic data. In particular, the number of double bonds found in GPs species seems to be yeast strain-specific and correlates well with the presence or absence of desaturase-encoding genes in the genome. This indicates that the methodology is not only useable to confirm genomic data, but can be rather useful in predicting the presence of desaturase-encoding genes. A GP profile could be a great contribution to construct pathways of lipid biosynthesis and metabolism. Consequently, the results show that profiling methods are important for further elucidation of the manifold roles of lipids.

To our knowledge, this work also presents, for the first time, a comprehensive GP profile of yeast strains other than the widely studied *S. cerevisiae*, some of which are appreciated for biotechnological or basic research purposes. The results could be an important first step for further elucidation of the roles of lipids in these particular strains, which would contribute significantly to new scientific perceptions.

## Supplementary Files

Supplementary File 1 XLS-Document (XLS, 83 KB)
